# Effects of Single-Nucleotide Polymorphisms in Cytokine, Toll-Like Receptor, and Progesterone Receptor Genes on Risk of Miscarriage

**DOI:** 10.1155/2018/9272749

**Published:** 2018-07-17

**Authors:** Anastasiia Razdaibiedina, Mykhaylo Khobzey, Victoria Tkachenko, Iryna Vorobiova

**Affiliations:** ^1^National Institute of Pediatrics, Obstetrics and Gynecology, National Academy of Medical Sciences of Ukraine, Kyiv, Ukraine; ^2^Department of Computer Science and Cybernetics, Taras Shevchenko National University of Kyiv, Kyiv, Ukraine

## Abstract

Spontaneous abortion is a complex, multifactorial pathology, where various genetic, neural, endocrine, and immunological factors are involved. Cytokines, Toll-like receptors, and progesterone receptors play critical roles in embryonic implantation and development. A delicate, stage-specific equilibrium of these proteins is required for successful pregnancy outcome. However, genetic variation from one individual to another results in variation in levels of Th1/Th2 cytokines, strength of identification of infectious agents by Toll-like receptors, and quality of progesterone recognition. Thus, a complex study encompassing effects of major SNPs of cytokine, TLR, and PGR genes on the risk of miscarriage is needed. In this study, we investigated SNPs of 9 genes (TLR2 G753A, TLR4 C399T, TLR9 G2848A, TGF-*β*1 C509T, PGR PROGINS, IL-6 G174C, IL-8 C781T, IL-10 C592A, and TNF*α* G308A) in 106 women, whose pregnancy ended in miscarriage, and 74 women, who delivered in term without any pregnancy complication. All participants are from Ukrainian population. As a result, TLR9 and IL-10 SNPs have been found to play critical roles in the development of spontaneous abortion. TLR2, TLR4, IL-6, IL-8, and PGR SNPs were identified as secondary factors that can also affect the risk of miscarriage. There was no association found between TGF-*β*1 and TNF*α* polymorphisms and miscarriage.

## 1. Introduction

One of the leading problems of obstetrics is the miscarriage of pregnancy, which occurs in up to 30% of all the desirable pregnancies and has no tendency to decrease [1, 2]. Even after conducting a thorough examination, in more than 50% of cases, the cause of pregnancy loss remains unknown, and therapeutic measures aimed at diagnosing the cause of miscarriage are not always effective [1]. Pregnancy loss is considered a multifactorial pathology [2–4], and study of the genetic factors that can act as prerequisites for miscarriage is a promising direction of personalized medicine aimed at prevention of various obstetric complications [5].

Normal performance of reproductive function in women is subject to the harmonious functioning of neural, immune, and endocrine mechanisms [6, 7]. Three major groups of proteins that regulate these processes and are responsible for cell signaling, immune responses, and controlling the course of pregnancy are cytokines, Toll-like receptors, and progesterone receptors, respectively [7–10].

It is generally accepted that preterm birth is stimulated by excessive production of proinflammatory cytokines such as TNF*α*, IL-8, and IL-6 that stimulate synthesis of prostaglandins—triggers of preterm birth [11], while anti-inflammatory cytokines such as IL-10 and TGF-*β*1 are involved in promoting the normal course of fetal development [12, 13]. However, the long-standing Th1/Th2 ratio paradigm is now considered an oversimplification, since it has been shown in previous studies that levels of Th1/Th2 cytokines vary from one woman to another during pregnancy [6, 9, 14]. This variation is mostly explained by individual genetic variation of cytokine encoding genes [9]. Thus, different cytokine genes' SNPs result in different levels of cytokine production and, accordingly, different strength of cytokine responses [6, 9]. That is why it is crucial to study the effects of cytokines' SNPs on the risk of spontaneous abortion. To encompass impact of important proinflammatory and anti-inflammatory cytokines' SNPs, we have chosen to study three Th1 cytokine genes—TNF*α*, IL-8, and IL-6, and two Th2 cytokine genes—IL-10 and TGF-*β*1.

Toll-like receptor single-nucleotide polymorphisms induce individual variation in the quality of identification of infectious agents during pregnancy [14–16]. Mutant TLR SNPs are often associated with imbalance in the system of innate immunity and, as a result, an increase in mother organism sensitivity to the infections and the development of chronic inflammatory processes, which increase the risk of miscarriage [14, 15]. We have included three important and different in function Toll-like receptors (TLR2, TLR4, and TLR9) to study the relation between TLR SNPs and risk of spontaneous abortion.

Variations in PGR gene expression are considered to affect the risk of pregnancy loss, but the relationship between SNPs of PGR gene and pregnancy outcome still remains unclear. That is why we have included common PGR polymorphism in our study of the risk of spontaneous abortion.

The aim of this study was to conduct broad research of genetic factors that contribute to the risk of spontaneous abortion, encompassing SNPs of cytokines, TLR, and PGR genes. This is the first study that investigates both individual effects of genes and effects of genetic interactions on the risk of miscarriage.

## 2. Materials and Methods

### 2.1. Population Studies

106 women with miscarriage and 74 women with a physiological course of pregnancy were monitored in obstetric clinics of the Institute of Pediatrics, Obstetrics and Gynecology of the National Academy of Medical Sciences of Ukraine in 2015–2017.

All participants underwent clinical and genetic investigation to ensure that no known etiologic factors of miscarriage occurred in either study or control group. All participants come from different regions of Ukraine and have Ukrainian ancestry. The control group consisted of 74 pregnant women in obstetric clinics of the Institute of Pediatrics, Obstetrics and Gynecology, who had no pregnancy complications and delivered in term. The study group consisted of 106 women, whose pregnancy ended in miscarriage.

The appropriate ethics committee approved this research project, and all the participants gave written consent.

### 2.2. Genotyping Procedures

To identify single-nucleotide polymorphisms (SNPs) of the studied genes, we used DNA samples isolated from nuclei of peripheral blood cells. The collection of biological material for laboratory research was conducted under condition of exclusion of any medical treatments during the period of no acute diseases and no remission of chronic somatic diseases for more than 1 month. Identification of polymorphic alleles of genes was carried out by the method of allele-specific polymerase chain reaction with subsequent electrophoretic detection on agarose gel.

In order to determine the effect of inflammatory reactions in the pathogenesis of miscarriage, genotyping of promoter sites was carried out using the following polymorphic markers: G753A for TLR2; C399T for TLR4; G2848A for TLR9; C509T for TGF-*β*1; PROGINS for PGR; G174C for IL-6; C781T for IL-8; C592A for IL-10; and G308A for TNF*α*.

### 2.3. Statistical Analysis

All statistical calculations were completed with software environment for statistical computing and graphics R (version 3.4.3).

We used Pearson's chi-squared test with level of significance *α*=0.05 for each of the studied 9 genes' polymorphisms to determine whether there is a significant difference between the observed genotype frequencies in women with miscarriage and genotype frequencies in general population. Genotype frequencies were derived by direct counting specific genotype occurrence in a group of interest and division by the total number of people in the group.

Considering the unknown effects of the studied genes' polymorphisms on the risk of miscarriage, we used both multiplicative and general additive models of inheritance to assess the *p* values and odds ratios (ORs) with 95% confidence intervals (CIs). If A is wild allele and B is mutant allele, multiplicative model assumes *k*-fold increased risk for AB and *k*^2^ increased risk for BB genotype, and analysis is performed by allele. On the contrary, general additive model assumes *k*-fold increased risk for AB and 2*k* increased risk for BB genotype, and analysis is performed by genotype with help of Armitage's test for trend [17].

To investigate combined effects of the studied genes' SNPs on the pregnancy outcome (e.g., miscarriage vs. normal pregnancy), we used multiple logistic regression model for binary dependent variable on a combined dataset of women, whose pregnancy ended in miscarriage, and women who delivered in term. *Z*-values were calculated by using the Wald test.

## 3. Results

### 3.1. Individual Genetic Contribution to the Risk of Miscarriage

**Figure 1:**
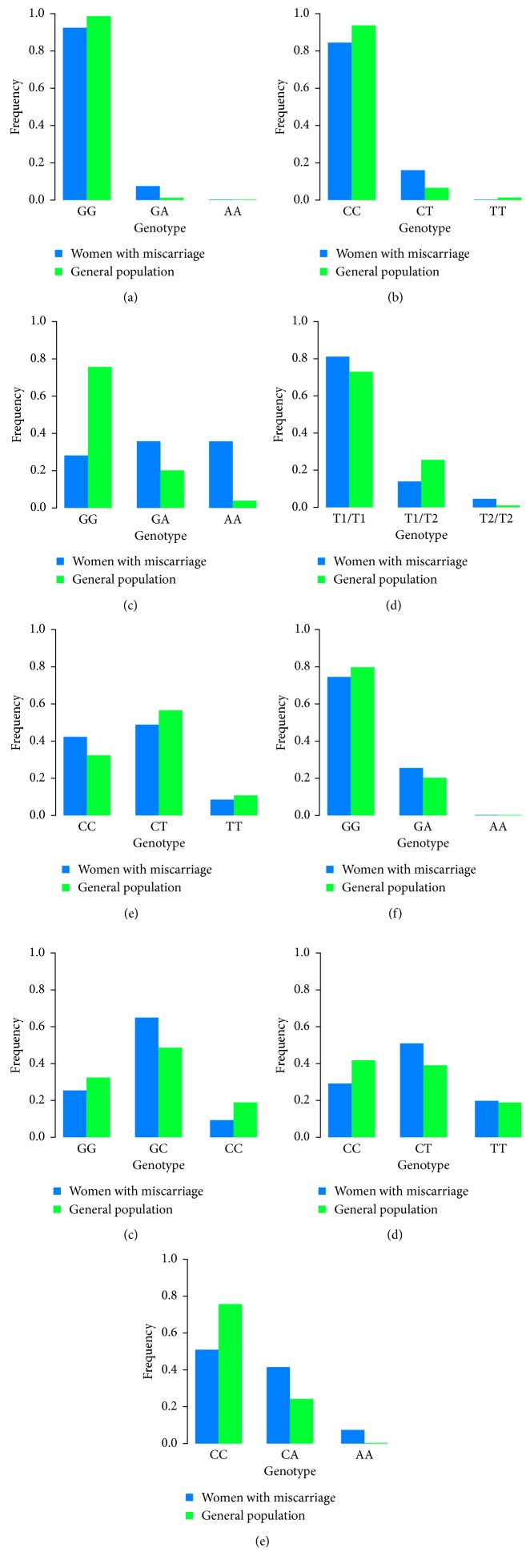
Frequencies of studied SNP genotype occurrence in women with miscarriage (blue) and general population (green). (a) TLR2 G753A, (b) TLR4 C399T, (c) TLR9 G2848A, (d) PGR PROGINS, (e) TGF-*β*1 C509T, (f) TNF*α* G308A, (g) IL-6 G174C, (h) IL-8 C781T, and (i) IL-10 C592A.

Firstly, we assessed individual effects of genes' SNPs on the risk of spontaneous abortion. We compared genotype and allele frequencies of each of 9 genes' SNPs (TLR2 G753A, TLR4 C399T, TLR9 G2848A, TGF-*β*1 C509T, PGR PROGINS, IL-6 G174C, IL-8 C781T, IL-10 C592A, and TNF*α* G308A) in 106 women with spontaneous abortion and in the control group of 74 women who delivered in term (Figure 1, Tables 1–18). To visualize differences in genotype frequencies, we built 9 grouped bar plots—one for each of the studied polymorphisms, with frequency of genotypes in women with miscarriage and general population, respectively (Figure 1).

Chi-squared test with 95% confidence interval identified two genes in the set of studied genes, whose SNP allele and genotype frequency difference in women with miscarriage and control group is statistically significant—TLR9 (*p* < 0.0001 for both multiplicative and additive models of inheritance) (Tables 5 and 6) and IL-10 (*p*=0.0003 for multiplicative model of inheritance and *p*=0.001 for additive model of inheritance) (Tables 15 and 16).

After calculating the odds ratios (CI = 95%) for the studied genes, we have found SNP alleles and genotypes that are associated with the risk of miscarriage.

The following SNP genotypes drastically increase the risk of spontaneous abortion: IL-10 AA (OR = 12.86, *p*=0.001) (Table 16), IL-10 CA (OR = 2.21, *p*=0.001) (Table 16), TLR9 AA (OR = 13.23, *p* < 0.0001) (Table 6), TLR9 GA (OR = 2.20, *p* < 0.0001) (Table 6), TLR2 GA (OR = 5.96, *p*=0.17) (Table 2), TLR4 CT (OR = 2.64, *p*=0.17) (Table 4), PGR PROGINS T2/T2 (OR = 3.61, *p*=0.09) (Table 8), and IL-6 GC (OR = 1.97, *p*=0.06).

The following SNP mutant alleles significantly increase the risk of spontaneous abortion: TLR9 A (OR = 7.03, *p* < 0.0001) (Table 5), IL-10 A (OR = 2.85, *p*=0.0003) (Table 15), TLR2 A (OR = 5.76, *p*=0.06) (Table 2), and TLR4 T(OR = 2.49, *p*=0.07) (Table 3).

### 3.2. Combined Effects of Genes on the Risk of Miscarriage

To assess combined effects of the studied genes' SNPs on the pregnancy outcome, we used multiple logistic regression model on categorical variables. Logistic regression enables us to estimate the probability of the binary outcome using the input values of the independent categorical variables [18].

Considering all genetic SNPs with a mutant allele as input variables, the following SNP genotypes were found to be significantly positively associated with the risk of miscarriage: TLR9 AA (*z* = 4.180, *p*=2.92*e*−05), TLR9 GA (*z* = 4.184, *p*=2.87*e*−05), IL-10 CA (*z* = 3.573, *p*=0.000353), TLR2 GA (*z* = 3.027, *p*=0.00247), and IL-8 CT (*z* = 2.862, *p*=0.004205). And IL-6 CC (*z* = −2.854, *p*=0.004313) genotype shows statistically significant, negative association with the risk of miscarriage (Table 19).

To study the combined effects of genes' SNPs associated with miscarriage, we tested these genes for pairwise and triple genetic interactions (combined effect of genes).

The following double genetic interactions showed statistically significant association with the risk of miscarriage: TLR9 and IL-6 (Table 20); TLR9 and IL-8 (Table 21); TLR9 and IL-10 (Table 22); and IL-10 and TLR4 (Table 23).

TLR 9 GG and IL-6 GG interaction (*z* = −3.078, *p*=0.002) and TLR 9 GG and IL-6 GC interaction (*z* = −2.8, *p*=0.005) had a statistically significant negative effect on the risk of miscarriage. TLR9 GG and IL-8 CC interaction (*z* = −2.797, *p*=0.005), TLR9 GA and IL-8 CC interaction (*z* = −2.615, *p*=0.0089), TLR9 GG and IL-8 CT interaction (*z* = −2.612, *p*=0.0089), and TLR9 GG and IL-8 TT interaction (*z* = −3.318, *p*=0.0009) had a statistically significant negative effect on the risk of miscarriage. TLR9 GG and IL-10 CC interaction (*z* = −3.418, *p*=0.0006) and TLR9 GG and IL-10 CA interaction (*z* = −3.094, *p*=0.0019) had a statistically significant negative effect on the risk of miscarriage. IL-10 AA and TLR4 CC interaction (*z* = 62.728, *p* < 0.0001) and IL-10 CA and TLR4 CT interaction (*z* = 62.728, *p* < 0.0001) had a statistically significant positive effect on the risk of miscarriage.

The following triple genetic interaction showed statistically significant association with the risk of miscarriage: TLR9, IL-10, and TLR4 (Table 24). TLR9 AA, IL-10 AA, and TLR4 CC interaction (*z* = 44.826, *p* < 0.001) and TLR9 GG, IL-10 CA, and TLR4 CT interaction (*z* = 44.826, *p* < 0.001) had a statistically significant positive effect on the risk of miscarriage.

We have outlined the effect on the risk of miscarriage of the major double and triple genotype interactions in Tables 25 and 26.

## 4. Discussion

In order to provide an accurate prognosis of the probability of miscarriage, it is crucial to account for the genetic characteristics of a patient. Since pregnancy is delicately balanced between inflammation and intrauterine infections, immune and endocrine factors should always be in stage-specific equilibrium to avoid pregnancy loss [19]. And hence, genetic variation in genes that encode proteins, which are involved in immune, endocrine, and neural mechanisms is an important factor at determining spontaneous abortion risk [6,7]. SNPs in cytokine, Toll-like receptor, and progesterone receptor genes can have severe impact on the pregnancy outcome [6, 7, 10]. Thus, it is very important to study individual effects of SNPs of these genes on the risk of spontaneous abortion. Moreover, it is very advantageous to take into account not only the influence of a single SNP in a gene, but also the combined effect of SNPs in several genes, because genetic interactions may yield new emergent properties, which cannot be attributed to any of the genes separately, but happen when specific genotypes occur together [20].

This is the first complex study of SNPs in genes of three important protein families (cytokines, TLRs, and PGRs) that play critical roles in pregnancy miscarriage. We investigated both individual and combined effects of genes' SNPs on the risk of pregnancy loss.

Chi-squared test results showed that SNPs in TLR9 and IL-10 genes have statistically significant effect on the risk of miscarriage with highest of all genes' ORs of 13.23 and 12.86, respectively, for double-mutant genotypes.

Although SNPs in TLR2, TLR4, PGR, and IL-6 did not exhibit statistically significant differences in genotype or allele frequencies between study and control groups, they had high values of ORs (>1.97) for either mutant allele or genotype.

Mutant SNPs in TLR9, IL-10, TLR2, IL-6, and IL-8 genes were found to have statistically significant effect on the risk of miscarriage with help of multiple logistic regression.

The multiple logistic regression analysis has also shown that the following double interactions showed statistically significant association with the risk of miscarriage: TLR9 and IL-10; TLR9 and IL-8; TLR9 and IL-6; and IL-10 and TLR4. Moreover, one triple genetic interaction was significantly associated with the risk of miscarriage: TLR9, IL-10, and TLR4.

Thus, TLR9 and IL-10 SNPs have been found to have the most significant effect on the risk of miscarriage, figuring as major factors of spontaneous abortion in all performed statistical tests. Also, TLR9 and IL-10 SNPs exhibited genetic interactions with statistically significant association with the risk of spontaneous abortion.

Previous studies have shown association between TLR9 G2848A polymorphisms and increased inflammation risk at the maternal-fetal interface in Caucasian population [21]. Our data provide further support to the hypothesis that SNPs in TLR9 gene can result in altered innate immunity and dramatically increase risk of inflammatory miscarriage. It has been previously shown that IL-10 deficiency contributes to infertility, spontaneous abortion, and other severe pregnancy pathologies [11]. Our data provide further evidence to the hypothesis that IL-10 SNPs that result in decreased IL-10 production lead to increased risk of miscarriage.

Next factors that can also be considered when predicting the risk of spontaneous abortion are SNPs in TLR2, TLR4, IL-6, IL-8, and PGR genes. Although these genes did not show strong association results in all statistical tests, they have been found to have a significant effect on the risk of miscarriage by some of the tests. Effects of SNPs in TLR2, IL-6, and IL-8 were identified as statistically significant by multiple logistic regression. IL-6, TLR2, TLR4, and PGR had high allele-wise or genotype-wise ORs. IL-6, IL-8, and IL-10 SNPs exhibit statistically significant genetic interaction with TLR9, while TLR4 SNP exhibits statistically significant genetic interaction with IL-10.

Previous studies have shown association between increased production of Th1 cytokines (including IL-6 and IL-8) and risk of miscarriage [22]. Our results further support previous findings, indicating that SNPs that increase IL-6 and IL-8 production can have adverse effects on pregnancy. Previous studies of TLR2 and TLR4 SNPs on the risk of spontaneous abortion yield controversial results [23–26]. Our findings suggest that TLR2 and TLR4 SNPs are associated with imbalance in the system of innate immunity and, as a result, an increase in mother's organism sensitivity to the infections and miscarriage risk. Previous research about PGR PROGINS polymorphism association with the risk of miscarriage is scarce and has controversial results: while some studies suggest that PGR gene mutant allele increases risk of recurrent spontaneous abortion [27], other studies suggest that there is no association between PGR gene SNPs and idiopathic spontaneous abortion [28]. Our data show that PGR PROGINS double-mutant genotype (T2/T2) can significantly increase the risk of spontaneous abortion.

Interestingly, previous studies have shown that TLR2 G753A SNPs are associated with urinary tract infection, TLR4 C399T SNPs are associated with various infections and Crohn's disease, and TLR9 G2848A SNPs are associated with cervical cancer [29].

There was no association found between TGF-*β*1 and TNF*α* polymorphisms and miscarriage.

## 5. Conclusions

We have conducted a complex study that encompasses effects of single-nucleotide polymorphisms in genes of three major protein families (cytokines, Toll-like receptors, and progesterone receptors) on the risk of spontaneous abortion in Ukrainian women.

TLR9 and IL-10 SNPs have been found to play critical roles in the development of spontaneous abortion. Moreover, these genes can increase the risk of miscarriage when their genotypes co-occur with other genotypes. This way, TLR9 and IL-10 exhibit double genetic interaction with each other, TLR9 interacts with IL-6 and IL-8, and IL-10 interacts with TLR4.

TLR2, TLR4, IL-6, IL-8, and PGR SNPs can also affect the risk of miscarriage, but in less extent than TLR9 and IL-10. There was no association found between TGF-*β*1 and TNF*α* polymorphisms and miscarriage.

To further elucidate and confirm the found effects of the studied polymorphisms on the risk of spontaneous abortion, there is a need to perform studies on larger and more heterogeneous population groups.

## Figures and Tables

**Table 1:** TLR2 G753A polymorphism analysis with multiplicative model of inheritance.

Allele	Women with miscarriage frequency	Control group frequency	*χ* ^2^	*p*	Odds ratio	
					Values	95% CI
G	0.962	0.994	3.43	0.06	0.17	0.02–1.40
A	0.038	0.007			5.76	0.71–46.59

Estimated allele frequencies as well as corresponding odds ratios and 95% confidence interval (significance level of 0.05) in the group of women with miscarriage and the control group.

**Table 2:** TLR2 G753A polymorphism analysis with general additive model of inheritance.

Genotype	Women with miscarriage frequency	Control group frequency	*χ* ^2^	*p*	Odds ratio	
					Values	95% CI
GG	0.925	0.986	3.52	0.17	0.17	0.02–1.37
GA	0.075	0.014			5.96	0.73–48.71
AA	0.000	0.000			0.70	0.01–35.65

Estimated genotype frequencies as well as corresponding odds ratios and 95% confidence interval (significance level of 0.05) in the group of women with miscarriage and the control group.

**Table 3:** TLR4 C399T polymorphism analysis with multiplicative model of inheritance.

Allele	Women with miscarriage frequency	Control group frequency	*χ* ^2^	*p*	Odds ratio	
					Values	95% CI
C	0.920	0.966	3.27	0.07	0.40	0.14–1.11
T	0.080	0.034			2.49	0.90–6.92

Estimated allele frequencies as well as corresponding odds ratios and 95% confidence interval (significance level of 0.05) in the group of women with miscarriage and the control group.

**Table 4:** TLR4 C399T polymorphism analysis with general additive model of inheritance.

Genotype	Women with miscarriage frequency	Control group frequency	*χ* ^2^	*p*	Odds ratio	
					Values	95% CI
CC	0.840	0.932	3.50	0.17	0.38	0.13–1.08
CT	0.160	0.068			2.64	0.93–7.50
TT	0.000	0.010			0.70	0.01–35.65

Estimated genotype frequencies as well as corresponding odds ratios and 95% confidence interval (significance level of 0.05) in the group of women with miscarriage and the control group.

**Table 5:** TLR9 G2848A polymorphism analysis with multiplicative model of inheritance.

Allele	Women with miscarriage frequency	Control group frequency	*χ* ^2^	*p*	Odds ratio	
					Values	95% CI
G	0.462	0.858	58.27	3.0E–12	0.14	0.08–0.24
A	0.538	0.142			7.03	4.12–12.01

Estimated allele frequencies as well as corresponding odds ratios and 95% confidence interval (significance level of 0.05) in the group of women with miscarriage and the control group.

**Table 6:** TLR9 G2848A polymorphism analysis with general additive model of inheritance.

Genotype	Women with miscarriage frequency	Control group frequency	*χ* ^2^	*p*	Odds ratio	
					Values	95% CI
GG	0.283	0.757	43.40	4.0E-10	0.13	0.06–0.25
GA	0.358	0.203			2.20	1.10–4.39
AA	0.358	0.041			13.23	3.90–44.87

Estimated genotype frequencies as well as corresponding odds ratios and 95% confidence interval (significance level of 0.05) in the group of women with miscarriage and the control group.

**Table 7:** PGR PROGINS polymorphism analysis with multiplicative model of inheritance.

Allele	Women with miscarriage frequency	Control group frequency	*χ* ^2^	*p*	Odds ratio	
					Values	95% CI
T1	0.882	0.858	0.45	0.5	1.24	0.66–2.30
T2	0.118	0.142			0.81	0.43–1.51

Estimated allele frequencies as well as corresponding odds ratios and 95% confidence interval (significance level of 0.05) in the group of women with miscarriage and the control group.

**Table 8:** PGR PROGINS polymorphism analysis with general additive model of inheritance.

Genotype	Women with miscarriage frequency	Control group frequency	*χ* ^2^	*p*	Odds ratio	
					Values	95% CI
T1/T1	0.811	0.730	4.92	0.09	1.59	0.79–3.23
T1/T2	0.142	0.257			0.48	0.22–1.02
T2/T2	0.047	0.014			3.61	0.41–31.59

Estimated genotype frequencies as well as corresponding odds ratios and 95% confidence interval (significance level of 0.05) in the group of women with miscarriage and the control group.

**Table 9:** TGF-*β*1 C509T polymorphism analysis with multiplicative model of inheritance.

Allele	Women with miscarriage frequency	Control group frequency	*χ* ^2^	*p*	Odds ratio	
					Values	95% CI
C	0.670	0.608	1.45	0.23	1.31	0.84–2.07
T	0.330	0.392			0.76	0.49–1.18

Estimated allele frequencies as well as corresponding odds ratios and 95% confidence interval (significance level of 0.05) in the group of women with miscarriage and the control group.

**Table 10:** TGF-*β*1 C509T polymorphism analysis with general additive model of inheritance.

Genotype	Women with miscarriage frequency	Control group frequency	*χ* ^2^	*p*	Odds ratio	
					Values	95% CI
CC	0.425	0.324	1.88	0.39	1.54	0.83–2.86
CT	0.491	0.568			0.73	0.40–1.33
TT	0.085	0.108			0.77	0.28–2.09

Estimated genotype frequencies as well as corresponding odds ratios and 95% confidence interval (significance level of 0.05) in the group of women with miscarriage and the control group.

**Table 11:** IL-6 G174C polymorphism analysis with multiplicative model of inheritance.

Allele	Women with miscarriage frequency	Control group frequency	*χ* ^2^	*p*	Odds ratio	
					Values	95% CI
G	0.580	0.568	0.06	0.81	1.05	0.69–1.61
C	0.420	0.432			0.95	0.62–1.45

Estimated allele frequencies as well as corresponding odds ratios and 95% confidence interval (significance level of 0.05) in the group of women with miscarriage and the control group.

**Table 12:** IL-6 G174C polymorphism analysis with general additive model of inheritance.

Genotype	Women with miscarriage frequency	Control group frequency	*χ* ^2^	*p*	Odds ratio	
					Values	95% CI
GG	0.255	0.324	5.71	0.06	0.71	0.37–1.37
GC	0.651	0.486			1.97	1.07–3.61
CC	0.094	0.189			0.45	0.19–1.07

Estimated genotype frequencies as well as corresponding odds ratios and 95% confidence interval (significance level of 0.05) in the group of women with miscarriage and the control group.

**Table 13:** IL-8 C781T polymorphism analysis with multiplicative model of inheritance.

Allele	Women with miscarriage frequency	Control group frequency	*χ* ^2^	*p*	Odds ratio	
					Values	95% CI
C	0.547	0.615	1.63	0.2	0.76	0.49–1.16
T	0.453	0.385			1.32	0.86–2.03

Estimated allele frequencies as well as corresponding odds ratios and 95% confidence interval (significance level of 0.05) in the group of women with miscarriage and the control group.

**Table 14:** IL-8 C781T polymorphism analysis with general additive model of inheritance.

Genotype	Women with miscarriage frequency	Control group frequency	*χ* ^2^	*p*	Odds ratio	
					Values	95% CI
CC	0.292	0.419	3.35	0.19	0.57	0.31–1.07
CT	0.509	0.392			1.61	0.88–2.94
TT	0.198	0.189			1.06	0.50–2.25

Estimated genotype frequencies as well as corresponding odds ratios and 95% confidence interval (significance level of 0.05) in the group of women with miscarriage and the control group.

**Table 15:** IL-10 C592A polymorphism analysis with multiplicative model of inheritance.

Allele	Women with miscarriage frequency	Control group frequency	*χ* ^2^	*p*	Odds ratio	
					Values	95% CI
C	0.717	0.878	13.38	0.0003	0.35	0.20–0.62
A	0.283	0.122			2.85	1.60–5.07

Estimated allele frequencies as well as corresponding odds ratios and 95% confidence interval (significance level of 0.05) in the group of women with miscarriage and the control group.

**Table 16:** IL-10 C592A polymorphism analysis with general additive model of inheritance.

Genotype	Women with miscarriage frequency	Control group frequency	*χ* ^2^	*p*	Odds ratio	
					Values	95% CI
CC	0.509	0.757	13.68	0.001	0.33	0.17–0.66
CA	0.415	0.243			2.21	1.14–4.26
AA	0.075	0.000			12.86	0.73–226.33

Estimated genotype frequencies as well as corresponding odds ratios and 95% confidence interval (significance level of 0.05) in the group of women with miscarriage and the control group.

**Table 17:** TNF*α* G308A polymorphism analysis with multiplicative model of inheritance.

Allele	Women with miscarriage frequency	Control group frequency	*χ* ^2^	*p*	Odds ratio	
					Values	95% CI
G	0.873	0.889	0.57	0.45	0.77	0.40–1.51
A	0.127	0.101			1.29	0.66–2.53

Estimated allele frequencies as well as corresponding odds ratios and 95% confidence interval (significance level of 0.05) in the group of women with miscarriage and the control group.

**Table 18:** TNF*α* G308A polymorphism analysis with general additive model of inheritance.

Genotype	Women with miscarriage frequency	Control group frequency	*χ* ^2^	*p*	Odds ratio	
					Values	95% CI
GG	0.745	0.797	0.66	0.42	0.36	0.36–1.52
GA	0.255	0.203			0.66	0.66–2.75
AA	0.000	0.000			0.70	0.01–35.65

Estimated genotype frequencies as well as corresponding odds ratios and 95% confidence interval (significance level of 0.05) in the group of women with miscarriage and the control group.

**Table 19:** Analysis of the individual effects of TLR2, TLR4, TLR9, TNF*α*, PGR, IL-6, IL-8, IL-10, and TGF-*β*1 genotypes on the risk of miscarriage with multiple logistic regression.

	Estimate	Std. error	*z* value	Pr (>|*z*|)
(Intercept)	1.93624	0.67065	−2.887	0.003888^*∗∗*^
TLR2 GA	4.91960	1.62537	3.027	0.002472^*∗∗*^
TLR4 CT	1.07696	0.71345	1.510	0.131167
TLR9 AA	3.25877	0.77968	4.180	2.92e−05^*∗∗∗*^
TLR9 GA	2.22891	0.53278	4.184	2.87e−05^*∗∗∗*^
PGR T1T2	−0.79836	0.54428	−1.467	0.142428
PGR T2T2	−0.14348	1.43022	−0.100	0.920088
TGF-*β*1 CT	−0.61884	0.44058	−1.405	0.160142
TGF-*β*1 TT	−0.78374	0.85355	−0.918	0.358509
IL-6 CC	−2.23917	0.78448	−2.854	0.004313^*∗∗*^
IL-6 GC	0.18254	0.47991	0.380	0.703677
IL-8 CT	1.65668	0.57878	2.862	0.004205^*∗∗*^
IL-8 TT	0.85917	0.62898	1.366	0.171950
IL-10 AA	16.43724	1354.17780	0.012	0.990315
IL-10 CA	1.91552	0.53608	3.573	0.000353^*∗∗∗*^
TNF GA	−0.05164	0.49134	−0.105	0.916304

Significance codes: 0 ‘^*∗∗∗*^' 0.001 ‘^*∗∗*^' 0.01 ‘^*∗*^' 0.05 ‘.' 0.1 ‘ ' 1.

**Table 20:** Analysis of the combined effects of TLR9 and IL-6 SNPs on the risk of miscarriage with multiple logistic regression.

	Estimate	Std. error	*z* value	Pr (>|*z*|)
(Intercept)	1.0609	0.3867	2.743	0.00609^*∗∗*^
TLR9 GG + IL-6 GG	−1.6587	0.5390	−3.078	0.00209^*∗∗*^
TLR9 AA + IL-6 GG	0.3254	0.8801	0.370	0.71156
TLR9 GA + IL-6 GG	0.3254	0.8801	0.370	0.71156
TLR9 GG + IL-6 CC	−18.6269	1251.0541	−0.015	0.98812
TLR9 AA + IL-6 CC	16.5052	1615.1039	0.010	0.99185
TLR9 GA + IL-6 CC	−1.0609	0.8060	−1.316	0.18808
TLR9 GG + IL-6 GC	−1.3745	0.4906	−2.802	0.00508^*∗∗*^
TLR9 AA + IL-6 GC	2.1172	1.0914	1.940	0.05240

Significance codes: 0 ‘^*∗∗∗*^' 0.001 ‘^*∗∗*^' 0.01 ‘^*∗*^' 0.05 ‘.' 0.1 ‘ ' 1.

**Table 21:** Analysis of the combined effects of TLR9 and IL-8 SNPs on the risk of miscarriage with multiple logistic regression.

	Estimate	Std. error	*z* value	Pr (>|*z*|)
(Intercept)	1.7918	0.7638	2.346	0.018978^*∗*^
TLR9 GG + IL-8 CC	−2.4277	0.8679	−2.797	0.005154^*∗∗*^
TLR9 AA + IL-8 CC	−0.1178	0.9895	−0.119	0.905252
TLR9 GA + IL-8 CC	−2.3979	0.9170	−2.615	0.008925^*∗∗*^
TLR9 GG + IL-8 CT	−2.1432	0.8204	−2.612	0.008990^*∗∗*^
TLR9 AA + IL-8 CT	16.7743	1684.1382	0.010	0.992053
TLR9 GA + IL-8 CT	0.5108	1.0646	0.480	0.631343
TLR9 GG + IL-8 TT	−3.5835	1.0801	−3.318	0.000908^*∗∗∗*^
TLR9 AA + IL-8 TT	16.7743	2465.3258	0.007	0.994571

Significance codes: 0 ‘^*∗∗∗*^' 0.001 ‘^*∗∗*^' 0.01 ‘^*∗*^' 0.05 ‘.' 0.1 ‘ ' 1.

**Table 22:** Analysis of the combined effects of TLR9 and IL-10 SNPs on the risk of miscarriage with multiple logistic regression.

	Estimate	Std. error	*z* value	Pr (>|*z*|)
(Intercept)	2.0794	0.7500	2.773	0.005561^*∗∗*^
TLR9 GG + IL-10 CC	−2.7238	0.7969	−3.418	0.000631^*∗∗∗*^
TLR9 AA + IL-10 CC	−0.7802	0.9933	−0.785	0.432230
TLR9 GA + IL-10 CC	−1.5533	0.8276	−1.877	0.060519
TLR9 AA + IL-10 AA	16.4866	2306.1011	0.007	0.994296
TLR9 GG + IL-10 CA	−2.6548	0.8580	−3.094	0.001973^*∗∗*^
TLR9 AA + IL-10 CA	16.4866	1496.3961	0.011	0.991209

Significance codes: 0 ‘^*∗∗∗*^' 0.001 ‘^*∗∗*^' 0.01 ‘^*∗*^' 0.05 ‘.' 0.1 ‘ ' 1.

**Table 23:** Analysis of the combined effects of IL-10 and TLR4 SNPs on the risk of miscarriage with multiple logistic regression.

	Estimate	Std. error	*z* value	Pr (>|*z*|)
(Intercept)	−4.756*e* + 13	7.255*e* + 13	−0.656	0.512
IL-10 CC + TLR4 CC	4.756*e* + 13	7.255*e* + 13	0.656	0.512
IL-10 AA + TLR4 CC	4.551*e* + 15	7.255*e* + 13	62.728	<2*e* − 16^*∗∗∗*^
IL-10 CA + TLR4 CC	4.756*e* + 13	7.255*e* + 13	0.656	0.512
IL-10 CC + TLR4 CT	4.756*e* + 13	7.255*e* + 13	0.656	0.512
IL-10 CA + TLR4 CT	4.551*e* + 15	7.255*e* + 13	62.728	<2*e* − 16^*∗∗∗*^

Significance codes: 0 ‘^*∗∗∗*^' 0.001 ‘^*∗∗*^' 0.01 ‘^*∗*^' 0.05 ‘.' 0.1 ‘ ' 1.

**Table 24:** Analysis of the combined effects of TLR9, IL-10, and TLR4 SNPs on the risk of miscarriage with multiple logistic regression.

	Estimate	Std. error	*z* value	Pr (>|*z*|)
(Intercept)	−1.340*e* + 13	1.008*e* + 14	−0.133	0.894
TLR9 GG + IL-10 CC + TLR4 CC	1.340*e* + 13	1.008*e* + 14	0.133	0.894
TLR9 AA + IL-10 CC + TLR4 CC	1.340*e* + 13	1.008*e* + 14	0.133	0.894
TLR9 GA + IL-10 CC + TLR4 CC	1.340*e* + 13	1.008*e* + 14	0.133	0.894
TLR9 AA + IL-10 AA + TLR4 CC	4.517*e* + 15	1.008*e* + 14	44.826	<2*e* − 16^*∗∗∗*^
TLR9 GG + IL-10 CA + TLR4 CC	1.340*e* + 13	1.008*e* + 14	0.133	0.894
TLR9 AA + IL-10 CA + TLR4 CC	1.340*e* + 13	1.008*e* + 14	0.133	0.894
TLR9 GA + IL-10 CA + TLR4 CC	1.340*e* + 13	1.008*e* + 14	0.133	0.894
TLR9 GG + IL-10 CC + TLR4 CT	1.340*e* + 13	1.008*e* + 14	0.133	0.894
TLR9 AA + IL-10 CC + TLR4 CT	1.340*e* + 13	1.008*e* + 14	0.133	0.894
TLR9 GA + IL-10 CC + TLR4 CT	1.340*e* + 13	1.008*e* + 14	0.133	0.894
TLR9 GG + IL-10 CA + TLR4 CT	4.517*e* + 15	1.008*e* + 14	44.826	<2*e* − 16^*∗∗∗*^
TLR9 AA + IL-10 CA + TLR4 CT	1.340*e* + 13	1.008*e* + 14	0.133	0.894

Significance codes: 0 ‘^*∗∗∗*^' 0.001 ‘^*∗∗*^' 0.01 ‘^*∗*^' 0.05 ‘.' 0.1 ‘ ' 1.

**Table 25:** Major double genetic interactions that affect the risk of miscarriage.

Increasing risk of miscarriage	Decreasing risk of miscarriage
TLR9 GA + IL-6 CC	TLR9 GG + IL-6 GG
TLR9 GA + IL-6 GC	TLR9 GG + IL-6 GC
TLR9 GA + IL-8 CT	TLR9 GG + IL-8 TT
TLR9 GA + IL-8 TT	TLR9 GG + IL-8 CT
TLR9 AA + IL-8 CT	TLR9 GA + IL-8 CC
TLR9 AA + IL-8 TT	TLR9 GG + IL-8 CC
TLR9 GA + IL-10 AA	TLR9 GG + IL-10 CC
TLR9 GA + IL-10 CA	TLR9 GG + IL-10 CA
TLR9 AA + IL-10 AA	
TLR9 AA + IL-10 CA	
IL-10 AA + TLR4 CC	
IL-10 CA + TLR4 CT	

**Table 26:** Major triple genetic interactions that affect the risk of miscarriage.

Increasing risk of miscarriage	Decreasing risk of miscarriage
TLR9 GA + IL-10 AA + TLR4 CC	TLR9 GG + IL-10 CC + TLR4 CC
TLR9 GA + IL-10 AA + TLR4 CT	TLR9 GG + IL-10 CA + TLR4 CC
TLR9 AA + IL-10 CA + TLR4 CT	
TLR9 AA + IL-10 CA + TLR4 CC	

## Data Availability

The data used to support the findings of this study are available from the corresponding author upon request.

## Abbreviations

CI: Confidence interval
IL: Interleukin
NK: Natural killers
OR: Odds ratio
PGR: Progesterone receptor
SNP: Single-nucleotide polymorphisms
TGF-*β*1: Transforming growth factor *β*1
Th: Helper T cells
Th1: Type 1 helper T cells
Th2: Type 2 helper T cells
TLR: Toll-like receptor
TNF: Tumor necrosis factor.
